# PGK1 as Predictor of CXCR4 Expression, Bone Marrow Metastases and Survival in Neuroblastoma

**DOI:** 10.1371/journal.pone.0083701

**Published:** 2013-12-20

**Authors:** Helen M. Ameis, Astrid Drenckhan, Katharina von Loga, Gabriele Escherich, Katharina Wenke, Jakob R. Izbicki, Konrad Reinshagen, Stephanie J. Gros

**Affiliations:** 1 Department of Pediatric Surgery, University Medical Center Hamburg-Eppendorf/Altona Children's Hospital, Hamburg, Germany; 2 Department of General, Visceral and Thoracic Surgery, University Medical Center Hamburg-Eppendorf, Hamburg, Germany; 3 Department of Pathology, University Medical Center Hamburg-Eppendorf, Hamburg, Germany; 4 Department of Pediatric Hematology and Oncology, University Medical Center Hamburg-Eppendorf, Hamburg, Germany; University of Alabama at Birmingham, United States of America

## Abstract

**Background and Aim:**

A close relationship between phosphoglycerate kinase 1 (PGK1) and the CXCR4/SDF1 axis (chemokine receptor 4/stromal cell derived factor 1) has been shown for several cancers. However, the role of PGK1 has not been investigated for neuroblastoma, and PGK1 might be a therapeutic target for this tumor entity. The aim of the current study was to evaluate the role of PGK1 expression in neuroblastoma patients, to determine the impact of PGK1 expression levels on survival, and to correlate PGK1 expression with CXCR4 expression and bone marrow dissemination.

**Materials and Methods:**

Samples from 22 patients with neuroblastoma that were surgically treated at the University Medical Center Hamburg-Eppendorf were evaluated for expression of PGK1 and CXCR4 using immunohistochemistry. Results were correlated with clinical parameters, metastases and outcome of patients. Immunocytochemistry, proliferation and expression analysis of CXCR4 and PGK1 were performed in neuroblastoma cell lines.

**Results:**

PGK1 is expressed in neuroblastoma cells. PGK1 expression is significantly positively correlated with CXCR4 expression and tumor dissemination to the bone marrow. Moreover the expression of PGK1 is significantly associated with a negative impact on survival in patients with neuroblastoma. PGK1 is downregulated by inhibition of CXCR4 in neuroblastoma cells.

**Conclusion:**

PGK1 appears to play an important role for neuroblastoma, predicting survival and tumor dissemination. Further in vivo studies outstanding, it is a candidate target for novel therapeutic strategies.

## Introduction

Neuroblastoma arises from sympathetic neuroblast cells derived from the neural crest and is the most frequent solid tumour in childhood outside the central nervous system [1], [2]. The tumor can combine characteristics of its originating cells, with extensive heterogeneity, pluripotential differentiation and migratory abilities, leading to wide range of clinical presentation from spontaneous regression to fatal progression and dissemination to privileged sites [3], [4]. The outcome strongly correlates with clinical factors (e.g. age, stage and chromosomal aberrations), but the overall survival of patients suffering from this tumour entity is good [5]. The prognosis of high-risk neuroblastoma with disseminated disease (International Neuroblastoma Staging System stage IV) is still poor [5]–[11].

Besides lymph node involvement, metastastic dissemination in advanced stages of highly malignant neuroblastoma occurs most frequently to bone marrow, bone, liver, and skin [6]–[12]. Metastastic homing involving the tumor cells as well as the target tissue still remains an unsolved and intriguing question [13], [14].

It has been proposed that the chemokine receptor CXCR4 is involved in the mechanisms by which neuroblastoma cells metastasize to specific sites [15], [16]. A higher expression of CXCR4 was found in primary neuroblastoma from patients with high-stage disease and in patients with bone marrow metastases. Clinical outcome in patients with high level expression of CXCR4 is significantly worse than in patients with low CXCR4 tumor expression [17]. It was suggested that neuroblastoma cell homing to the bone marrow is influenced by the various interactions of the chemokine receptor CXCR4 and its ligand, the stromal cell-derived factor-1 (SDF1) [18]–[23].

It has been shown, that during angiogenesis SDF1 signalling reduces the expression and secretion of phosphoglycerate kinase 1 (PGK1) [24], [25]. PGK1 is an ATP-generating glycolytic enzyme that forms part of the glycolytic pathway [26] and is regulated by hypoxia-inducible factor-1α (HIF-1α) [27]. Extracellular PGK1 facilitates the cleavage of plasminogen generating the vascular inhibitor angiostatin [28]–[30], which is known as an important regulator of an “angiogenic switch” [25]. This determines a close relationship between the regulation of the CXCR4/SDF1 axis and PGK1 in prostate cancer [25]. PGK1 also appears to be a crucial enzyme for peritoneal dissemination of gastric cancer in both CXCR4/SDF1-dependent and by CXCR4/SDF1-independent mechanisms, making high levels of PGK1 essential for tumor growth and metastasis and showing a direct relationship between PGK1 signalling and CXCR4 [31], [32]. These findings support the importance of cross-talk between glucose metabolism and chemokine function. This axis might serve as potential therapeutic option [31], [32]. Although the interaction of the CXCR4/SDF1-PGK1 axis to our knowledge has not been researched for neuroblastoma, PGK1 might be a possible therapeutic target also for this tumor entity.

The aim of the current study was to evaluate the role of PGK1 expression in neuroblastoma patients, to determine the impact of PGK1 expression levels on survival, and to correlate PGK1 expression with CXCR4 expression and bone marrow dissemination.

## Materials and Methods

### Patients

Samples from 22 patients with neuroblastoma that were surgically treated at the University Medical Center Hamburg-Eppendorf between Juli 2005 and Oktober 2011 were used for this study. Tumor samples were selected on the basis of availability of tissues and follow-up data.

Clinical follow-up data were obtained by reviewing the hospital records, contacting patients on an outpatient basis or by phone call. Overall survival was calculated from the date of surgery to the date of death or last follow-up. None of the patients died from a cause other than neuroblastoma. All tumours were categorised into groups according to the International Neuroblastoma Staging System (INSS) [1]. Histological grading was determined according to Hughes [33]. None of the patients had been pretreated. The study was approved by the Ethics Committee of the Chamber of Physicians in Hamburg, Germany. Written informed consent was obtained from all parents of the patients for the use of the resected samples and clinical data for research purposes.

### Immunohistochemistry

For the immunhistochemistry the HRP-ACE-System from R&D Systems (Mineapolis, USA) were used. Sections were counterstained with Mayer's hematoxylin solution (Merck). Tumor tissue was identified by hematoxylin eosin (HE) staining. The CXCR4 staining was performed using the primary rabbit polyclonal CXCR4 antibody (Abcam, clone 2074, UK) at a dilution of 1∶250. The PGK1 staining was performed using the primary mouse polyclonal PGK1 antibody (Santa Cruz Biotechnology, sc-48342, USA) at a dilution of 1∶200. As control sections were incubated with antibody diluent (DAKO, Denmark) without primary antibody at 4°C overnight and then treated as other samples. The immunostaining was scored by two examiners.

### Immunocytochemistry, proliferation and western blot analysis

Neuroblastoma Kelly cells (Sigma-Aldrich, Munich, Germany) and SH-EP Tet-21/N cells (reported by Lutz et al.[34], [35], kindly provided by G. Eschenburg, Hamburg) were cultivated in RPMI media containing 10% FCS and seeded in Chamber Slides (BD-Bioscience, Falcon, USA). After cultivation at 37°C and 7% CO2 for 24 h the cells were fixated with 4% paraformaldehyd and washed thrice with PBS-Tween solution. Following blocking of unspecific binding in 3% BSA solution cells were incubated overnight at 4°C with the primary antibodies for PGK1 and CXCR4 (see Immunohistochemistry) or antibody diluent as control. Following washing the cells were incubated with the secondary antibody labelled with Fitc (AlexFlour 488) at room temperature for 1 h, washed and mounted with fluorescence mounting media with DAPI (Dako, Denmark).

Cell proliferation under treatment with AMD3100 was examined with the Cell Titer 96® Aqueous one solution cell proliferation assay MTT-Assay (Promega Corporation). In a 96 well plate 5.000 cells per well were plated in RPMI-media supplemented with 10% FCS and incubated at 37°C with 5%CO2. After 24 h incubation cells received either 20 µg AMD3100 (Sigma-Aldrich, Munich, Germany) or PBS as control. 48 h after stimulation the MTT-assay were performed according to manufacture's protocol and the absorbance was measured in an enzyme-linked immunosorbent assay reader (Microplate Reader, Dynatech MR500). Absorbance was related to the starting concentration. The experiment was repeated at least twice.

Kelly and SH-EP Tet-21/N cells were treated equally for 48 h with AMD3100 and PBS respectively for protein isolation for western blot analysis. Cell lysates were prepared in RIPA-Puffer (Sigma-Aldrich) combined with 100x Halt-Protease-Inhibitor Cocktail (Thermo-Scientific). The protein concentration was determined using BCA-Protein-Assay Kit (Thermo-Scientific). Protein (40 µg) from each cell line and treatment were mixed with Laemmli-Puffer (5% SDS, 20% Glycerine, 4% β-Mercaptoethanol in Tris 0,5 M, pH 6.8, and 1% Bromphenoleblue) treated for 5 min at 96°C. The samples were loaded on a 10% SDS-Page. After separation the proteins were electrotransferred to a nitrocellulose membrane (Thermo-Scientific). The proteins were immunoblotted using Anti-PGK-1 (Santa Cruz, sc-23802, in a dilution of 1∶500). The bands were detected using the Super Signal West Dura Extended Duration Substrate (Thermo-Scientific) For re-blotting, membranes were stripped according to manufacture's protocol. An anti-Tubulin antibody (Cellsignaling, in a dilution of 1∶3000) served as a control.

### Bone marrow analysis

Bone marrow samples were obtained from four different locations (the anterior and posterior superior iliac crests on both sides) of neuroblastoma patients at the time of initial diagnosis. Bone marrow smears were immediately placed on glass slides. Visualization was achieved by Giemsa staining. Stained slides were evaluated by two independent examiners, who came to congruent result in all cases, using standard microsopy. The occurrence of metastatic cells in at least one location was classified as bone marrow involvement.

### Statistical analysis

The statistical analysis was conducted using SPSS version 13.0 (SPSS, Chicago, IL, USA). A p-value less than 0.05 was defined as significant. For correlation of PGK1 expression with CXCR4 expression, overall metastases and bone marrow metastases the Spearman's-rank-correlation-coefficient was used. Kaplan-Meier survival analysis and log-rank test were performed to compare the survival time between groups. For *in vitro* experiments, the two-sided t-test was used for significance testing. Normal distribution of the measured values was proven before. The errorbars in the barplots represent the standard deviation.

## Results

### Patient characteristics

In total, 22 surgically resected pediatric neuroblastoma specimens were included in this study.

The mean age of the patients at the time of operation was 711 days, the mean follow up time 1194 days. Staging and grading are summarized in Table 1. Metastases had occurred in 59% of the patients. Bone marrow dissemination was found in 36% of the patients.

**Table 1:** Patient characteristics.

		All patients	PGK1+	PGK1-
		n = 22	n = 7 (31.8%)	n = 15 (68.2%)
**Age at operation**	d (mean)	14–2372 (711.14)	39–2373 (1112.71)	14–1516 (523.73)
**Follow up time**	d (mean)	105–2351 (1194.23)	105–2351 (1194.23)	135–2179 (1260.27)
**INSS stage (%)**	**1**	6 (27.3)	1(14.3)	5 (33.3)
	**2**	0 (0)	0 (0)	0 (0)
	**3**	5 (22.7)	1(14.3)	4 (26.7)
	**4**	7 (31.8)	4 (57.3)	3 (20)
	**4 s**	4 (18.2)	1 (14.3)	3 (20)
**Hughes grade (%)**	**1 a/b**	3 (13.6)	1 (14.3)	2 (13.3)
	**2**	5 (22.7)	0 (0)	5 (33.3)
	**3**	14 (63.6)	3 (85.7)	8 (53.3)
**Metastases**	**Positive**	13 (59,1)	6 (85,7)	7 (46.7)
n patients (%)	**Negative**	9 (40,9)	1 (14,3)	8 (53.3)
**Bone Marrow**	**Positive**	8 (36.4)	5 (71.4)	3 (20)
n patients (%)	**Negative**	14 (63.6)	2 (28.6)	12 (80)
**CXCR4**	**Positive**	10 (45.5)	6 (85.7)	4 (26.7)
n patients (%)	**Negative**	12 (54.5	1 (14.3)	11 (73.3)
**Survival**	**Dead**	4 (18.2)	4 (75,1)	0 (0)
n patients (%)	**Alive**	18 (81.8)	3 (42,9)	15 (100)

### PGK1 expression in neuroblastoma

**Figure 1:**
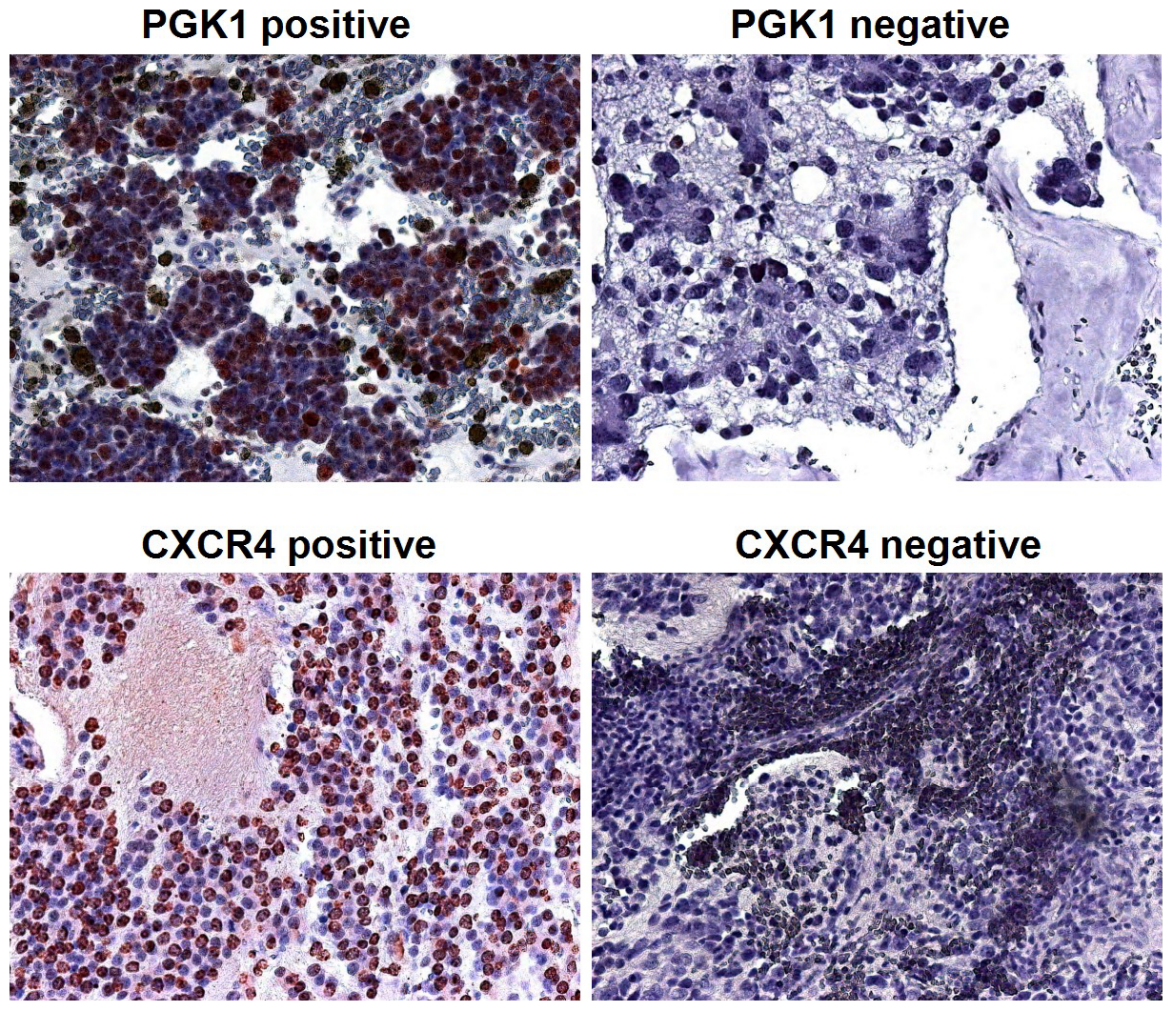
Immunohistochemistry for PGK1 and CXCR4. Representative images of PGK1 and CXCR4 positive and negative immunohistochemical staining of neuroblastoma tissue are shown (20x standard microscopic enlargement).

PGK1 expression of the 22 neuroblastoma specimens was determined by immunohistochemistry. Fig. 1 shows representative staining patterns for PGK 1. Lack of staining or weak staining of PGK 1 (i.e., ≤20% of tumor cells expressed PGK) was classified as PGK1-negative expression and moderate to strong staining (i.e., >20% of tumor cells expressed PGK1) was classified as PGK1-positive expression. A total of 7 (32%) of the 22 tumors were PGK1 positive and 15 (68%) samples were PGK1 negative (Table 1).

### PGK1 and CXCR4 expression

**Figure 2:**
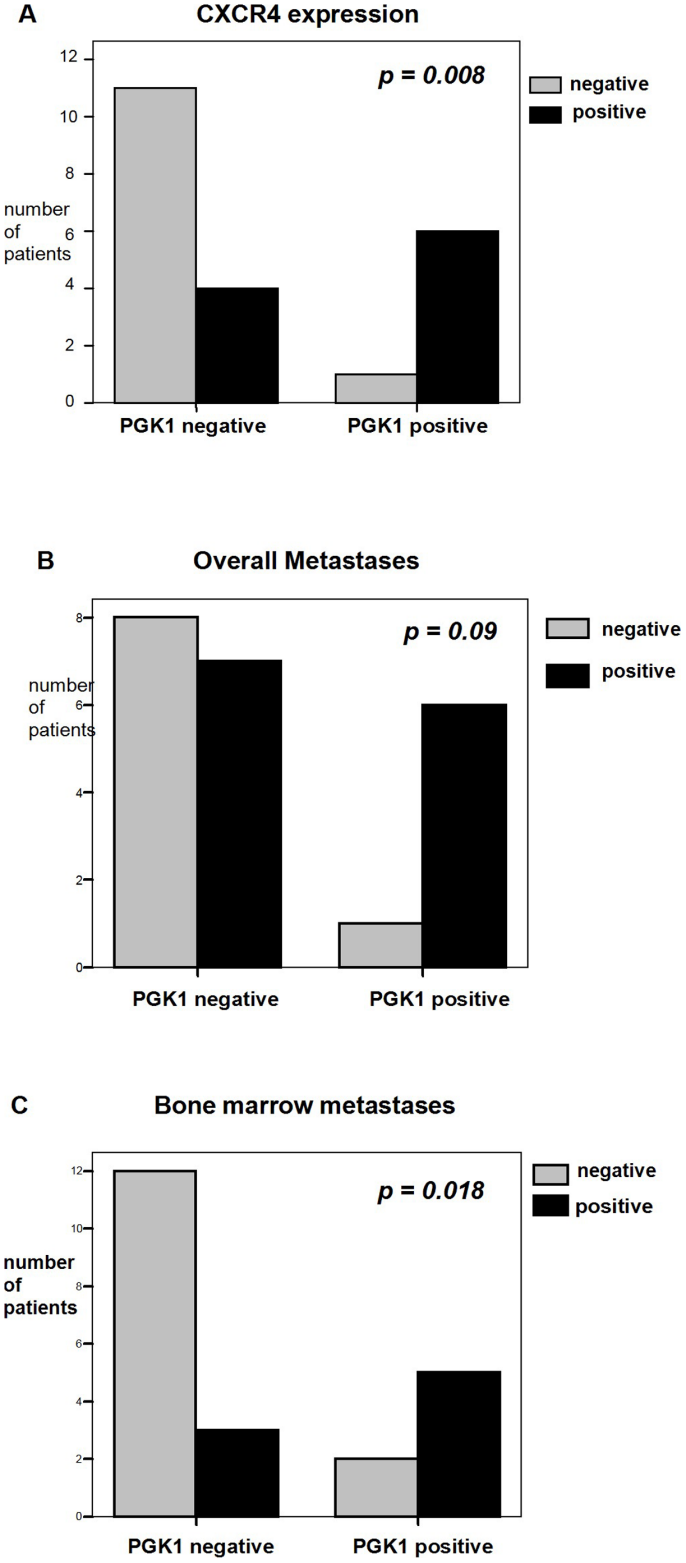
Correlation of PGK1 expression with CXCR4 expression, overall metastases and bone marrow metastases. **A** Significant positive correlation of positive PGK1 expression with positive CXCR4 expression depicted in a bar chart (*correlation coefficient 0.522, p = 0.008*). **B** The bar chart depicts a positive correlation of positive PGK1 expression with overall metastases without reaching the significance level of 0.05 (*correlation coefficient 0.379, p = 0.09*). **C** Significant positive correlation of positive PGK1 expression with bone marrow dissemination depicted in a bar chart (*correlation coefficient 0.498, p = 0.018*).

CXCR4 expression of the tumor specimen was determined by immunohistochemistry. Representative staining patterns for CXCR4 were shown in Figure 1. Classification of CXCR4 expression was performed analogous to the PGK1 expression classification mentioned above. A total of 10 (45%) of the 22 tumors were CXCR4 positive and 12 (55%) samples were CXCR4 negative (Table 1). Of the 10 CXCR4 positive tumors 6 (86%) expressed PGK1, while of the 12 CXCR4 negative cases only 1 (14%) showed expression of PGK1. A significant positive correlation of positive PGK1 and positive CXCR4 expression could be observed (*correlation coefficient 0.522, p = 0.008*) (Figure 2a).

### PGK1 and bone marrow dissemination

All 22 patients were examined for bone marrow metastases. Bone marrow dissemination was found in 8 (36%) of the patients (Table 1). In total, metastases were found in 13 patients (59.1%), which besides bone marrow mainly occurred in lymph nodes and liver (Table 2). When examining these 13 metastatic cases (overall metastases), 6 (86%) tumors expressed PGK1, while of the 9 (41%) not metastasised cases only 1 (14%) showed expression of PGK1 indicating an association. However a correlation of overall metastases with expression of PGK1 was not significant (*correlation coefficient 0.379, p = 0.09*) (Figure 2b). There was also no correlation of overall metastases with the expression of CXCR4 (*correlation coefficient 0.941, p = 0.937*).

**Table 2:** Metastatic sites.

Metastatic site (n = 13 patients)	number of patients	percentage of patients (%)
Lymph nodes	7	31.8
Liver	6	27.3
Bone marrow	8	36.4
Bone	1	4.5
Continuous spread to pancreas	1	4.5
Continuous spread to muscle	1	4.5

Of the 8 patients with bone marrow dissemination 5 (71%) tumors expressed PGK1, while of the 14 bone marrow negative cases only 2 (29%) showed expression of PGK1 (Table 1). A significant positive correlation of positive PGK1 and bone marrow dissemination could be observed (*correlation coefficient 0.498, p = 0.018*) (Figure 2c).

### Impact of PGK1 on survival

**Figure 3:**
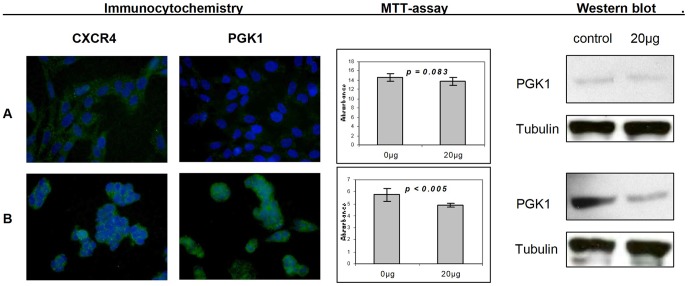
Overall Survival. For Kaplan-Meier survival analysis patients were grouped according to positive and negative PGK1 expression. All patients that died during the follow-up period showed positive PGK1 expression, while none of the PGK1 negative patients died. Overall survival of neuroblastoma patients with a PGK1 negative expression was significantly better than that of PGK1 positive patients (*p = 0.003*).

We next examined the relationship between PGK1 expression and survival of patients with neuroblastoma. Overall survival was analyzed by the Kaplan-Meier method, and the log-rank test was used for univariate analysis (Figure 3). PGK1-positive expression in the primary tumor was statistically significantly associated with poorer overall survival than PGK1-negative expression (*p = 0.003*). All 4 (18%) patients that died during the follow-up period showed PGK1-positive expression of the tumor, while of the 18 living patients only 3 showed positivity for PGK1.

### PGK1 and CXCR4 expression of neuroblastoma cell lines

**Figure 4:**
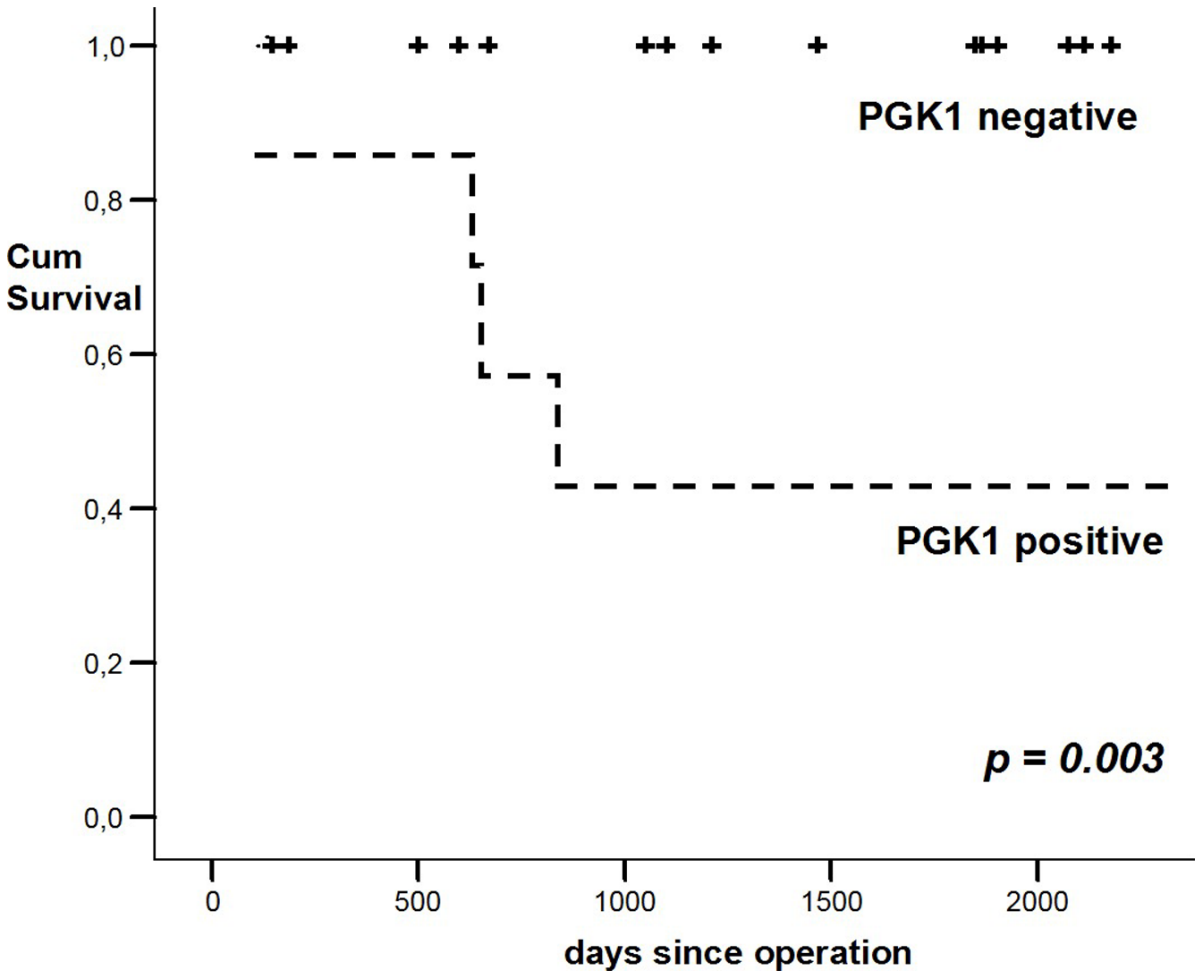
PGK1 and CXCR4 expression, proliferation and inhibition of CXCR4 of neuroblastoma cell lines. Kelly (**A**) and SH-EP Tet-21/N (**B**) neuroblastoma cells were immunostained for PGK1 and CXCR4 expression (**Immunohistochemistry**). Both cell lines show a positivity for CXCR4 and react to treatment with 20 µg AMD3100 with an inhibition of proliferation (**MTT-assay**), although only SH-EP Tet-21/N cells reach a significant level of growth reduction. On examination of PGK1 protein expression levels (**Western blot**) after 48 h of CXCR4 receptor inhibition, treatment with 20 µg AMD3100 leads to a downregulation of PGK1 protein (45 kDa) in SH-EP Tet-21/N but not in Kelly cells. Tubulin (55 kDa) served as control.

Kelly and SH-EP Tet-21/N neuroblastoma cell lines were examined for expression of PGK1 and CXCR4. Kelly cells showed a strong expression of CXCR4, while SH-EP Tet-21/N cells not only showed a strong CXCR4 expression but also expression of PGK1 (Figure 4). Inhibition of the CXCR4 receptor with 20 µg AMD3100 led to an inhibition of proliferation, although only SH-EP Tet-21/N cells reached a significant level of growth reduction. This suggests that the role of CXCR4 for cell proliferation might depend on the simultaneous overexpression of PGK1. On examination PGK1 expression levels in western blotting 48 h after inhibition of the CXCR4 receptor, Kelly cells retained the weak expression of PGK1, while SH-EP Tet-21/N cells downregulated their formerly strong expression of the PGK1 protein to a moderate level. This is an indicator for a functional linkage between the CXCR4 receptor pathway and PGK1.

## Discussion

We report here, to our knowledge for the first time, the expression of PGK1 in neuroblastoma. PGK1 expression significantly positively correlates with CXCR4 expression, which is known to be an important player in the tumor biology of neuroblastoma, and tumor dissemination to the bone marrow. Moreover the expression of PGK1 is significantly associated with a negative impact on survival in patients with neuroblastoma.

### Role of PGK1 expression

The expression and secretion of the ATP-generating glycolytic enzyme PGK1 has been shown to be involved in several physiological mechanism, most importantly angiogenesis and glucose metabolism, but also metastases under the regulation of SDF1 and HIF-1α amongst others [24], [25], [27]. A close relationship between the regulation of the CXCR4/SDF1 axis and PGK1 has been shown for prostate cancer [25], influencing the “angiogenic switch” [25] through the regulation of angiostatin [28]–[30]. PGK1 also appears to be a crucial enzyme for peritoneal dissemination of gastric cancer [31], [32]. Here, high levels of PGK1 seem to be essential for tumor growth and metastasis. However, the role of PGK1 has not been investigated for neuroblastoma, and PGK1 could well be a novel therapeutic target for this tumor entity. In our current study we describe not only the expression of PGK1 in about a third of the tumors, but also a negative impact of the occurrence of PGK1 on survival. This demonstrates the relevance of PGK1 also for neuroblastoma. As the small sample size of this retrospective study is the main limitation, prospective studies should be performed in the future to include more patient material with immunohistochemical staining of bone marrow samples in an attempt to confirm the results described here. The downregulation of PGK1 by inhibition of the CXCR4 receptor indicates a functional linkage of their pathways. Although further *in vitro* and *in vivo* studies targeting PGK1 should be conducted, the present results suggest that PGK1 may be a promising therapeutic target.

### Interaction of PGK1 with the CXCR4/SDF1 axis

A close relationship between PGK1 and the CXCR4/SDF1 axis is known for several cancers [36]–[44]. CXCR4 is a seven-transmembrane G-protein-coupled chemokine receptor. Together with its ligand SDF1 it promotes progression and metastatatic homing of a number of malignant diseases including prostate, non–small-cell lung, pancreatic, breast, gastric and esophageal cancer [36]–[44]. In the studies on gastric cancer a direct relationship between PGK1 signalling and CXCR4 is observed and supports the importance of interaction between glucose metabolism and chemokine function [31], [32]. Therefore the authors conclude that the CXCR4/SDF1-PGK1 axis might serve as a potential therapeutic target [31], [32]. Our *in vitro* results suggest that in neuroblastoma cells CXCR4 in combination with overexpression of PGK1 also plays a role in cell proliferation. The positive correlation of PGK1 and CXCR4 expression in neuroblastoma patients as well as the downregulation of PGK1 through inhibition of the CXCR4 receptor, which we found in our in vitro studies, are indicators for a causal interaction between the PGK1 pathway and the CXCR4/SDF1 axis.

### Dissemination to the bone marrow

Although, in spite of extensive research, the mechanisms of metastasis and tumor cell homing are still poorly understood, it is known that certain types of cancer preferentially metastasize to particular sites [45]. A major preferential metastatic site for several types of cancer, including breast and prostate carcinoma as well as neuroblastoma is the bone marrow [3], [7], [8], [10], [11], [46], [47]. In neuroblastoma, dissemination to the bone marrow can be detected in approximately 65% of children diagnosed with stage IV disease using a standard morphologic examination of aspirates and biopsies [8], [11]. Patients with very high risk disease can be identified by quantification of neuroblastoma cells in bone marrow [8], [11].

In addition to its critical role in tumor cell growth, survival and angiogenesis in multiple cancers, the CXCR4/SDF1 axis has been shown to mediate homing and metastatic secondary growth in SDF1-producing organs, such as liver and bone marrow [24], [48], [49]. It was hypothesised that the CXCR4 receptor participates in the metastatic homing of tumor cells to the bone marrow through secretion of SDF1 by the bone marrow stromal cells [20], [38]. It also has been shown that the CXCR4 receptor plays a role in the bone metastasis of prostate carcinoma [42], and in the bone marrow metastasis of myeloma and neuroblastoma cells [15], [50]. Recent reports indicate that CXCR4 is commonly expressed on neuroblastoma metastases in the bone marrow and that it may be actively contributing to neuroblastoma tumor cell homing to the bone marrow [16], [51]. However, the role of the CXCR4/SDF1 axis in the complex processes of organ-specific dissemination has been strongly debated [12], [51]–[54]. For example, an in vitro study of neuroblastoma cell lines as well as patient samples, CXCR4 could not demonstrate a functional role, although it was expressed on bone marrow metastases [52]. A further study found that the CXCR4/SDF1 axis strongly enhances cell growth without increasing in vivo invasion in neuroblastoma progression [12]. This makes PGK1 an interesting independent indicator for tumor cell dissemination.

CXCR4 expression is increased by HIF-1α [55], and a study revealed higher expression of HIF-1α in breast carcinomas of patients with bone marrow metastasis [56]. Whether HIF-1α has a role in the metastasis of neuroblastoma cells is yet to be determined. However, HIF-1α plays an important role in the regulation of PGK1 [27], and makes further functional investigation desirable. The positive correlation of PGK1 with bone marrow metastases combined with the negative impact on survival we found in our study is an indicator that PGK1 might serve as an independent factor in the complex homing of neuroblastoma cells to the bone marrow.

### Conclusion

The expression of PGK1 is significantly associated with a negative impact on survival and tumor dissemination to the bone marrow in patients with neuroblastoma. PGK1 expression positively correlates with CXCR4 expression in neuroblastoma patients and is downregulated by inhibition of CXCR4 in neuroblastoma cells. Our data indicate that PGK1 plays an important role in neuroblastoma tumor growth and dissemination. Further in vivo studies outstanding, it is a candidate target for novel therapeutic strategies.

## Ackowledgments
